# Counseling for opioids prescribed at discharge of hospitalized adolescent trauma patients

**DOI:** 10.1186/s40621-023-00465-2

**Published:** 2023-10-23

**Authors:** Michael J. Mello, Lois K. Lee, Emily Christison-Lagay, Anthony Spirito, Sara Becker, Julie Bromberg, Stephanie Ruest, Mark R. Zonfrillo, Kelli Scott, Charles Pruitt, Karla Lawson, Isam Nasr, Jeremy Aidlen, Janette Baird

**Affiliations:** 1 Injury Prevention Center of Rhode Island Hospital-Hasbro Children’s Hospital, 593 Eddy Street, Claverick Building, Providence, RI 02903 USA; 2 Division of Emergency Medicine, Boston Children’s Hospital, 333 Longwood Avenue, Boston, MA USA; 3 Yale Pediatric Surgery, Yale New Haven Children’s Hospital, 1 Park Street, New Haven, CT USA; 4 Department of Psychiatry and Human Behavior, Alpert Medical School of Brown University, 222 Richmond Street, Providence, RI USA; 5 Feinberg School of Medicine Center for Dissemination and Implementation Science, Northwestern University, 633 North St Clair, 20th Floor, Chicago, IL USA; 6 Pediatric Emergency Medicine, Primary Children’s Hospital, 100 N Mario Capecchi Dr, Salt Lake City, UT USA; 7 Dell Children’s Trauma and Injury Research Center, Trauma Services, Dell Children’s Medical Center of Central Texas, 4900 Mueller Blvd., Austin, TX USA; 8 Division of Pediatric Surgery, Johns Hopkins Children’s Center, The Johns Hopkins Hospital, 1800 Orleans St. The Charlotte R. Bloomberg Children’s Center Building, Suite 7323, Baltimore, MD USA; 9 Division of Pediatric Surgery, UMass Memorial Medical Center - University Campus, 55 Lake Avenue North, Worcester, MA USA

**Keywords:** Prescription opioids, Adolescent, Pediatric trauma centers

## Abstract

**Background:**

Expert consensus recommends prescription opioid safety counseling be provided when prescribing an opioid. This may be especially important for youth with preexistent alcohol and other drug (AOD) use who are at higher risk of developing opioid use disorder. This study examined the frequency that adolescent trauma patients prescribed opioids at hospital discharge received counseling and if this differed by adolescents’ AOD use.

**Method:**

This study was embedded within a larger prospective stepped-wedge type III hybrid implementation study of AOD screening across a national cohort of pediatric trauma centers. Data were collected during 2018–2021 from admitted adolescent trauma patients (12–17 yo) at seven centers. Patient data were extracted from the electronic health record (EHR) on any prescribed discharged opioids, documentation of counseling delivered on prescribed opioid, who delivered counseling, and patients’ AOD screening results. Additionally, adolescents received an online survey within 30 days of hospital discharge that included asking about hospital discussions on safe use of prescription pain medication.

**Results:**

Of the 247 adolescent trauma patients enrolled, 158 completed the 30-day survey. AOD screening results were documented in the EHR for 139 patients (88%), with 69 (44.1%) screening AOD-positive. Opioids at discharge were prescribed to 86 (54.4%) adolescent patients, with no significant difference between those screened AOD-positive and AOD-negative (42.4% vs. 46.3%, *p* = 0.89). Counseling was documented in the EHR for 30 (34.9%) of those prescribed an opioid and was not significantly different by sex, age, race, ethnicity or between adolescent patients with documentation of AOD use (29.3%) versus those who did not (33.3%, *p* = 0.71). According to the adolescent survey, among those prescribed an opioid, 61.2% reported someone had talked with them about safe use of newly prescribed pain medications with again no difference between AOD-positive and AOD-negative screening results (*p* = 0.34).

**Conclusions:**

Although adolescent trauma patients recalled discussions on safe use of prescribed pain medication more often than was documented in the EHR, these discussions were not universal and did not differ if adolescents had screened positive or negative for AOD use as documented in the EHR.

*Trial Registry*: clinicaltrials.gov NCT03297060.

## Background

The 2019 Youth Risk Behavior Survey found that 7.2% of responding high school students admitted to current prescription opioid misuse (Jones et al. 2019). Additionally, among students misusing opioids, frequent co-use of alcohol (59.4%) and marijuana (43.5%) was reported. Other research has found that among adolescents with prescription opioid misuse, 25.4% received opioids via the healthcare system (usually from a single provider) (Hudgins et al. 2019). We have previously reported that approximately half (53.5%) of injured adolescent patients admitted to ten pediatric trauma centers were prescribed an opioid at discharge with wide variability between centers in prescribing patterns (28.6–72%) (Mello et al. 2022). Misuse of prescription opioids among adolescents predicts future development of an opioid use disorder and is associated with a range of preventable health consequences, including death from accidental overdose (McCabe et al. 2007; Bhatia et al. 2020). For this reason, identifying and providing a preventive intervention to adolescents at risk of prescription opioid misuse is a public health priority.

It is currently not known how often counseling on safe use of prescription pain medications is delivered to those adolescents who are prescribed pain medications at discharge after an inpatient admission to a pediatric trauma center. Although not specific for pediatric surgical trauma patients, expert consensus across clinical guidelines and publications recommends prescription opioid safety counseling be provided when prescribing an opioid for surgical patients (Dowell et al. 2022; Torres et al. 2017; Kelley-Quon et al. 2021). In addition, some states require documentation of this counseling in the patient’s medical record to ensure requirements are being met (Rhode Island Department of State 2022). Prescription opioid safety counseling is especially important for pediatric trauma patients with preexistent alcohol and other drug (AOD) use, as studies have demonstrated that these youth are at significantly higher risk of developing an opioid use disorder (OUD) (Thrul et al. 2021; Whiteside et al. 2016).

Our primary research objective was to examine the frequency with which adolescent trauma patients prescribed opioids at discharge received counseling about safe opioid usage by trauma center staff, and our secondary objective was to evaluate whether rates of counseling differed for adolescents with AOD use, compared to those without AOD use, given the increased risk of OUD among this population.

## Methods

This is a secondary analysis of data that were embedded within a larger multicenter prospective stepped-wedge trial that evaluated an implementation model for AOD screening across a national cohort of pediatric trauma centers (clinicaltrials.gov NCT03297060) (Mello et al. 2018). Data utilized in this analysis were collected from 2018 to 2021 at seven pediatric trauma centers, prior to implementing a screening, brief intervention, and referral to treatment protocol for adolescent trauma patients at each of these study sites. During the first wave of the COVID-19 pandemic in 2020, recruitment was suspended for six months (March–September 2020) due to institutional infection control policies. A single-center institutional review board at the coordinating center, Lifespan IRB, approved the study protocol. Strengthening the Reporting of Observation Studies in Epidemiology guidelines were used to present this observational study (von Elm et al. 2007).

To facilitate data collection, research staff at each study site were trained by the coordinating center on the study’s electronic health record (EHR) data extraction protocol and reviewed the EHR of all admitted adolescent (12–17 years) patients with traumatic injuries at their site during the study period. The Principal Investigator at each site also independently abstracted data as a quality check from 10% of the EHRs, and this was monitored by the study’s coordinating center for interrater reliability. Any data discrepancies were identified by the study’s coordinating center and returned to the sites for final resolution. Each site provided de-identified study data that were collected and managed using REDCap electronic data capture tools hosted at the coordinating center. Patient data extracted from the EHR included age, gender, race and ethnicity, any prescribed discharged opioids, documentation of counseling received about opioids prescribed at hospital discharge, who delivered counseling (if applicable), and patients’ alcohol and drug screening results (toxicology data or verbal questionnaires). For the purposes of this analysis, if a pediatric trauma center reported that the adolescent had been screened for AOD with a toxicology screen or any validated questionnaire (e.g., CRAFFT, S2BI), and the results were positive, the adolescent was classified as AOD-positive.

Additionally, a convenience sample of adolescents (12–17 years) admitted to the trauma service were recruited to participate in an online survey sent by the coordinating center within 30 days of their hospital discharge. Each site was asked to recruit one hospitalized adolescent per month. Consistent with the design of the larger study, adolescents who screened positive for alcohol or other drug use, either by biologic testing or by interview screening by the clinical trauma staff, were oversampled. One parent or guardian provided consent, and the adolescent provided assent to participate. Adolescents who were non-English or non-Spanish speaking, in police custody, unable to cognitively understand the consent/assent process, under review by child protection services or hospitalized for a suicide attempt were excluded. The 30-day post-discharge online survey asked whether a doctor, nurse or social worker spoke with the adolescent about safe use of any newly prescribed pain medication during their hospitalization.

Data from the EHR and patient survey were matched and integrated for analysis. Patient demographic data obtained from the EHR, including sex (male vs. female), race (White, Black, Asian), ethnicity (Hispanic vs. non-Hispanic), and age (continuous and categorical, 12–14 years, ≥ 15 years), were summarized as mean (with standard deviation, SD) or percentages. Frequencies in the primary outcome of interest, whether counseling was provided for adolescents prescribed opioids, were calculated (0 = no documented counseling as per EHR vs. 1 = counseling documented), and differences in rates of counseling between those who screened negative versus positive for AOD were assessed, using Fishers exact and Chi-square tests. Data were analyzed by the coordinating center using SAS (version 9.4, Carey, NC).

## Results

**Fig. 1:**
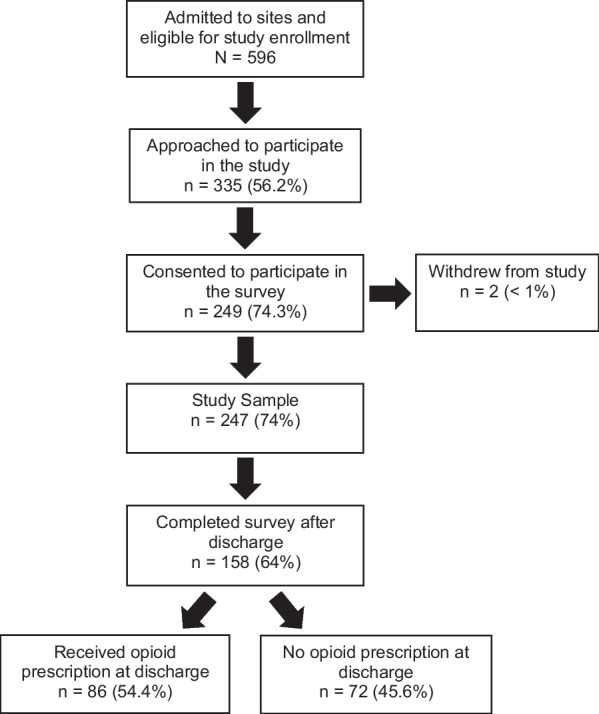
Participant enrollment, distribution, and retention

The participant enrollment flow across the seven participating trauma centers is depicted in Fig. 1. Of the 247 adolescent trauma patients recruited, 158 (64%) adolescents completed the 30-day survey and were included in this analysis. The respondents’ demographic characteristic data were taken from the EHR and are listed in Table 1.

**Table 1:** Characteristics of adolescent survey respondents

	Respondents *N* = 158
Gender *n* (%)	
Male	107 (69.9)
Race *n* (%)	
White	106 (67.1)
Black/African American	21 (13.3)
Asian	4(2.5)
Other	18 (11.4)
> 1 race	2 (< 1)
Refused/missing	11 (7)
Ethnicity *n* (%)	
Hispanic	23 (14.6)
Age mean (SD) range (years)	15.2 (1.7) 12–17.8

Of the 158 adolescents who completed the 30-day survey, 139 (88%) had been screened for AOD use according to the ER data. Positive AOD screens were recorded in the EHR for 69 patients (44.1%), and a positive screen was most frequently detected by the validated screening questions (*n* = 42, 60.9%) followed by both screening questions and biologic testing (*n* = 15, 21.7%), and biological testing only (*n* = 12, 17.4%).

Opioid medications were documented in the EHR as prescribed at discharge to 86 (54.4%) adolescent patients: 80 adolescents were prescribed oxycodone, 5 were prescribed hydrocodone, 1 was prescribed both, and 1 was prescribed another opioid medication. There was no significant difference between those who screened AOD-positive or AOD-negative in receiving an opioid prescription at discharge from the trauma center (42.4% vs. 46.3%, *p* = 0.89).

### Primary aim outcome

Counseling on safe prescription opioid use was documented in the EHR for 30 (34.9%) of the 86 patients prescribed an opioid. This counseling was documented as predominantly being provided by a nurse (66.7%) or a resident physician (23.3%). None of the opioid counseling in the EHR was documented as being provided by social work, addiction medicine, psychology or psychiatry staff. Additionally, EHR-documented delivery of opioid counseling did not significantly differ by patient sex (*p* = 0.52), age (*p* = 0.20), race (*p* = 0.73), or ethnicity (*p* = 0.38).

Contradictions were noted when the EHR documentation was compared to the 30-day online adolescent survey. Of those prescribed an opioid medication at discharge and providing survey data on counseling received (*n* = 85), 52 adolescents (61.2%) reported they had been counseled on safe use of their prescribed pain medication. Adolescent reports of who provided the counseling were much broader than what was documented in the EHR: 31(36.5%) by a physician/nurse and social worker, 16 (18.8%) from a physician/nurse only, and 5 (5.9%) from a social worker only. Further, 14 patients (16.5%) reported they did not know if they had received this counseling, with the remaining 19 patients (22.4%) reporting they had not received any counseling on safe prescription pain medication use.

### Secondary aim outcome

Among the 86 adolescents who were prescribed an opioid medication at discharge, there was no significant difference in EHR-documented opioid counseling between adolescent patients who reported AOD use (29.3%) versus those who did not (33.3%, *p* = 0.71). Additionally, among patients who had both survey data on safe prescription pain medication counseling and EHR documentation of AOD screening and opioid prescription(s) at discharge (*n* = 77), there was no significant difference in reports of receiving opioid safety counseling between those AOD-positive (24/36, 66.7%) and AOD-negative (23/41, 56.1%, *p* = 0.34). However, there were significant differences in the source of counseling with significantly more AOD-positive adolescents reported receiving opioid safety counseling from a social worker (55.6%) compared to the AOD-negative adolescents (33.3%, *p* = 0.04).

## Discussion

This multicenter cohort of adolescents receiving treatment in pediatric trauma centers examined the frequency with which patients prescribed opioids at discharge received counseling about safe opioid usage, and secondarily whether rates of counseling differed for adolescents with AOD use relative to those without AOD use. We found that more than half of adolescent trauma patients were prescribed an opioid pain medication at discharge (Mello et al. 2022). Despite expert consensus recommendations that youth prescribed an opioid receive universal counseling on opioid safety to prevent a future opioid use disorder, such counseling was only documented as occurring in about one of three patients in our sample. Even more concerning, there was no difference in the frequency of prescribing in those who screened positive compared to those screening negative for AOD use. Contrary to the EHR-documented data, when surveyed after discharge about safe prescription pain medication counseling, more adolescents recalled receiving some counseling (61.2%), and adolescents recalled receiving counseling from a broader range of clinicians. However, such counseling was far from consistent with expert consensus recommendations promoting universal screening, particularly among those adolescents with positive AOD screens who are at the highest risk for developing an OUD.

Although guidelines have been developed for opioid prescribing for pain management after pediatric surgery, adaptation of these guidelines for trauma patient aftercare, along with models for implementing universal opioid counseling within pediatric trauma center care, is both currently needed. Expert guidelines suggest that patient and caregiver counseling on safe opioid use should include the following: encourage nonopioid pain medication as the first line of treatment, use the minimum opioid dose necessary for pain management, consider nonpharmacologic pain management techniques, store medications safely (e.g., locked away) and properly dispose of unused opioid medication at the end of treatment (Mello et al. 2018).

In the current study, counseling was most often provided by nursing according to the EHR data. However, self-report interview data indicated that this counseling was commonly provided by a combination of nursing/physician and social worker. The varying roles of providers conducting counseling could be interpreted as positive because patients were getting the message from multiple sources. On the other hand, if counseling was not occurring universally, it suggests that no provider group had completely adopted opioid counseling as part of their workflow, potentially increasing the likelihood that adolescents would not receive any counseling related to prescribed opioid pain medication prior to hospital discharge. Use of social work consults may be an important model to implement for adolescents who screen positive for AOD use during admission to ensure the adolescent receives both a brief intervention for AOD use and integrate education on risks and safe practices for opioid pain medication that may be prescribed at discharge.

Additional work implementing comprehensive educational counseling to adolescent patients prescribed opioid pain medication is needed to identify opportunities and barriers for counseling, the optimal model for implementation and its impact on the prevention opioid misuse and opioid overdose prevention after discharge. A recent qualitative study of Canadian pediatric emergency physicians identified nine themes that influence physician opioid prescribing to youth (Slim et al. 2023), which included characteristics of the practice setting, physician confidence in medical evidence, personal biases and experiences, patient and family perspectives, opioid safety concerns, personal practice context, and media influences. Similar research is needed to identify factors influencing physicians’ decisions whether or not to provide opioid counseling, particularly in cases when the adolescent screens positive for AOD use.

Our study had several limitations that have the potential to affect our conclusions. First, we used documentation of counseling in the EHR as a primary outcome, which is at risk for misclassification bias. It is plausible that additional counseling was delivered by a provider during the adolescent’s hospitalization but was not documented. In fact, we found that more adolescents reported receiving counseling than was documented in the EHR. Second, we found that some (16.5%) youth could not recall if counseling was delivered, perhaps reflecting their cognitive state at the time of hospitalization, which raises the possibility that we are under-reporting actual counseling rates. The responses provided in the 30-day post-discharge surveys are subject to potential recall as well as responder biases. Third, we did not ask adolescents what instructions on safe opioid administration they were given during their counseling session, which limits our ability to ascertain the quality and completeness of the counseling received and whether the counseling affected adolescents’ safe use of opioids after discharge. We also did not assess providers’ knowledge, skills, or capacity to provide counseling and whether that influenced if counseling occurred. Furthermore, although our study recruited throughout the study period, we did not have 24/7 research staff coverage, did not recruit during the first 6 months of the COVID-19 pandemic, and 26% of approached eligible adolescents declined participation. This may have resulted in a nonrepresentative sample of adolescent admissions. Finally, our study was conducted in a convenience sample of level 1 pediatric trauma centers, which may also affect the generalizability of our results to other pediatric trauma centers and other healthcare settings.

## Conclusions

Results of this secondary analysis of a multicenter study of hospitalized adolescent trauma patients highlight an opportunity to promote universal opioid safety counseling among adolescents discharged with an opioid prescription. We found that more than half of the patients in our sample were prescribed an opioid pain medication at discharge. Although adolescents recalled counseling occurring more often than was documented, provision of safe opioid counseling was not universal. Concerningly, neither receipt of an opioid prescription nor opioid counseling differed based upon whether adolescents had screened positive or negative for AOD use, despite AOD use being a well-established predictor of a future opioid use disorder. Future work should evaluate implementation strategies to promote universal opioid safety counseling in pediatric trauma centers, particularly among youth screening positive for AOD, and examine the content and quality of those discussions.

## Data Availability

The datasets used and/or analyzed during the current study are available from the corresponding author on reasonable request.

## Abbreviations

AOD: Alcohol and other drugs
OUD: Opioid use disorder
EHR: Electronic health record
